# Supplementary material for: Genetic Analysis of QTL for Resistance to Maize Lethal Necrosis in Multiple Mapping Populations
Source: Genes (Basel). 2019 Dec 26;11(1):32. doi: 10.3390/genes11010032 (PMC7017159; doi:10.3390/genes11010032)
Supplement: Supplementary file 1 [file genes-11-00032-s001.pdf]

Table S1. Genetic linkage groups constructed for resistance to MLN using 500 SNPs in seven F_3 populations.

	**LG1**	**LG2**	**LG3**	**LG4**	**LG5**	**LG6**	**LG7**	**LG8**	**LG9**	**LG10**	**Total**
**CKDHL120918 × CML494**											
No. markers	13	15	13	14	11	12	5	15	5	9	112
Map length (cM)	190	146.28	64.08	174.75	109.04	141.43	90.19	93.8	86.62	127.79	1223.97
Ave. distance (cM)	14.61	9.75	4.93	12.48	9.91	11.79	18.04	6.25	17.32	14.2	10.93
**CML543 × CML494**											
No. markers	17	14	14	10	13	15	5	9	8	11	116
Map length (cM)	175.7	129.54	121.98	122.74	119.72	110.72	127.8	97.64	118.9	109.35	1234.11
Ave. distance (cM)	10.33	9.25	8.71	12.27	9.21	7.38	25.56	10.85	14.87	9.94	10.64
**CKDHL120918 × CML543**											
No. markers	19	9	9	9	16	17	4	16	7	12	118
Map length (cM)	78.13	137.03	131.69	121.61	120.5	121.72	55.42	139.13	53.8	140.24	1099.27
Ave. distance (cM)	4.11	15.23	14.63	13.51	7.53	7.16	13.86	8.7	7.69	11.69	9.32
**CKLTI0227 × CKDHL120918**											
No. markers	48	22	39	38	39	26	15	23	23	25	298
Map length (cM)	209.5	163.97	172.15	175.1	191	118.77	164.1	113.22	136.9	105.45	1550.07
Ave. distance (cM)	4.36	7.45	4.41	4.61	4.9	4.57	10.94	4.92	5.95	4.22	5.2
**CKDHL0089 × CKDHL120918**											
No. markers	17	15	17	12	21	14	4	13	10	5	128
Map length (cM)	179.3	171.83	124.36	78.7	147.42	133.94	42.92	135.64	102.1	65.6	1181.87
Ave. distance (cM)	10.55	11.46	7.32	6.56	7.02	9.57	10.73	10.43	10.21	13.12	9.23
**CKDHL221 × CKDHL120312**											
No. markers	19	15	16	12	14	10	3	13	5	11	118
Map length (cM)	196.9	119.97	128.13	128.15	114.04	65.79	4.56	126.19	40.18	146.01	1069.92
Ave. distance (cM)	10.36	8	8.01	10.68	8.15	6.58	1.52	9.71	8.04	13.27	9.07
**CKDHL0089 × CML494**											
No. markers	20	13	10	8	16	19	4	8	10	8	116
Map length (cM)	183.4	52.44	87.67	104.71	115.4	110.85	68.55	236.97	129.8	113.7	1203.51
Ave. distance (cM)	9.17	4.03	8.77	13.09	7.21	5.83	17.14	29.62	12.98	14.21	10.38
Total No. markers	153.00	103.00	118.00	103.00	130.00	113.00	40.00	97.00	68.00	81.00	1006.00

Overall mean	21.86	14.71	16.86	14.71	18.57	16.14	5.71	13.86	9.71	11.57	143.71
Total map length (cM)	1212.93	921.06	830.06	905.76	917.12	803.22	553.54	942.59	668.30	808.14	8562.72
Overall mean	173.28	131.58	118.58	129.39	131.02	114.75	79.08	134.66	95.47	115.45	1223.25
Total distance (cM)	63.49	65.17	56.78	73.20	53.93	52.88	97.79	80.48	77.06	80.65	64.77
Overall mean	9.07	9.31	8.11	10.46	7.70	7.55	13.97	11.50	11.01	11.52	9.25

Ch1–Chr10 = number of chromosomes.

Table S2. Twenty-three common QTLs and specific SNP detected in both linkage mapping and joint linkage association mapping across seven F_3 populations and their associated candidate genes.

SNP_Name	Chr	Position	QTL_Name	Fav Parent	Putative Candidate Gene	Predicted Function of Candidate Gene
PZA00447_8	1	9024005	*qMLN1_9*	CKDHL120312	GRMZM2G000039	Serine/threonine-protein phosphatase
PHM5622_21	1	183831657	*qMLN1_184*	CKDHL120918	GRMZM2G113726	Protein binding
S3_48493677	3	48493677	*qMLN3_48*	-	GRMZM2G017520	Transcription factor, Myb superfamily
S3_55444954	3	55444954	*qMLN3_55*	CKDHL120918	AC200298.4_FG001	Metal ion binding
S3_68596995	3	68596995	*qMLN3_68*	CKDHL120918	GRMZM2G132222	Ubiquitin-dependent protein catabolic process
PHM15449_10	3	125077922	*qMLN3_125*	CKDHL0089	GRMZM2G085433	Nucleic acid binding
S3_148291047	3	148291047	*qMLN3_148*	CKDHL120918	GRMZM2G156019	Phosphor lay signal transduction system
S3_151342843	3	151342843	*qMLN3_151*	CKDHL120918	GRMZM2G030902	Nucleic acid binding
PZA00920_1	3	142821031	*qMLN3_142*	CKDHL0221	GRMZM2G445999	Calcium ion binding
PZA00726_8	4	60767943	*qMLN4_60*	CKDHL120918	GRMZM2G030768	Zinc ion binding, hydrolase activity
S4_155378923	4	155378923	*qMLN4_155*	CKDHL120918	GRMZM2G137029	Protein binding
S5_170164477	5	170164477	*qMLN5_170*	CKDHL120918	GRMZM2G092588	Glycosyl transferases group
PHM7908_25	5	191075472	*qMLN5_191*	CML494	GRMZM2G097846	P-loop containing nucleoside triphosphate hydrolases
S5_196017729	5	196017729	*qMLN5_196*	-	GRMZM2G037200	Protein binding
S5_202816906	5	202816906	*qMLN5_202*	CKDHL120918	GRMZM2G480620	Uncharacterized protein
PHM563_9	5	204993639	*qMLN5_204*	CML494	GRMZM5G824439	Uncharacterized protein
S6_86475982	6	86475982	*qMLN6_86*	CKDHL120918	GRMZM2G083022	Protein binding; PPR: pentatricopeptide repeat domain
S6_89823772	6	89823772	*qMLN6_90*	CKDHL120918	GRMZM2G045414	-
PHM5235_8	8	94414978	*qMLN8_94*	CKDHL0221	GRMZM2G142427	-
S8_102533570	8	102533570	*qMLN8_102*	CKDHL0221	GRMZM5G805387	Transcription factor activity, sequence-specific DNA binding
PZA01313_2	10	3598262	*qMLN10_4*	CKDHL120918	GRMZM2G069382	LRR containing protein
PHM5740_9	10	8773358	*qMLN10_9*	CKDHL120918	GRMZM2G001272	Tub family proteins; protein binding
PZA00866_2	10	124203565	*qMLN10_124*	CML494	AC203841.3_FG002	DYW family of nucleic acid deaminases
